# Expression profiles analysis identifies specific interferon-stimulated signatures as potential diagnostic and predictive indicators of *JAK2V617F* ^+^ myelofibrosis

**DOI:** 10.3389/fgene.2022.927018

**Published:** 2022-08-18

**Authors:** Yanhong Zhao, Di Wang, Yipeng Liang, Changlu Xu, Lihong Shi, Jingyuan Tong

**Affiliations:** State Key Laboratory of Experimental Hematology, National Clinical Research Center for Blood Diseases, Haihe Laboratory of Cell Ecosystem, Institute of Hematology & Blood Diseases Hospital, Chinese Academy of Medical Sciences & Peking Union Medical College, Tianjin, China

**Keywords:** Myelofibrosis, *JAK2V617F*, MPN, Interferon (IFN), Prediction

## Abstract

**Objective:** This study aimed to identify specific dysregulated genes with potential diagnostic and predictive values for *JAK2V617F* ^+^ myelofibrosis.

**Methods:** Two gene expression datasets of CD34^+^ hematopoietic stem and progenitor cells (HSPCs) from patients with *JAK2V617F* ^+^ myeloproliferative neoplasm (MPN) [*n* = 66, including polycythemia vera (PV), essential thrombocythemia (ET), and primary myelofibrosis (PMF)] and healthy controls (HC) (*n* = 30) were acquired from the GEO (Gene Expression Omnibus) database. The differentially expressed genes (DEGs) were screened between each *JAK2V617F* ^+^ MPN entity and HC. Subsequently, functional enrichment analyses, including Kyoto Encyclopedia of Genes and Genomes (KEGG), Reactome, and Gene Set Enrichment Analysis (GSEA), were conducted to decipher the important biological effects of DEGs. Protein–protein interaction (PPI) networks of the DEGs were constructed to identify hub genes and significant modules. Another two gene expression profiles of patients with *JAK2V617F* ^+^ MPN [*n* = 23, including PV, ET, secondary myelofibrosis (SMF), and PMF] and HC (*n* = 6) from GEO were used as external validation datasets to prove the reliability of the identified signatures.

**Results:** KEGG analysis revealed the upregulated genes in three *JAK2V617F* ^+^ MPN entities compared with HC were essentially enriched in inflammatory pathways and immune response signaling pathways, and the number of these pathways enriched in PMF was obviously more than that in PV and ET. Following the PPI analysis, 10 genes primarily related to inflammation and immune response were found upregulated in different *JAK2V617F* ^+^ MPN entities. In addition, Reactome enrichment analysis indicated that interferon signaling pathways were enriched specifically in PMF but not in PV or ET. Furthermore, several interferon (IFN)-stimulated genes were identified to be uniquely upregulated in *JAK2V617F* ^+^ PMF. The external datasets validated the upregulation of four interferon-related genes (*OAS1*, *IFITM3*, *GBP1*, and *GBP2*) in *JAK2V617F* ^+^ myelofibrosis. The receiver operating characteristic (ROC) curves indicate that the four genes have high area under the ROC curve (AUC) values when distinguishing *JAK2V617F* ^+^ myelofibrosis from PV or ET.

**Conclusion:** Four interferon-stimulated genes (*OAS1*, *IFITM3*, *GBP1*, and *GBP2*) exclusively upregulated in *JAK2V617F* ^+^ myelofibrosis might have the potential to be the auxiliary molecular diagnostic and predictive indicators of myelofibrosis.

## Introduction

Polycythemia vera (PV), essential thrombocythemia (ET), and primary myelofibrosis (PMF) are a range of heterogeneous hematopoietic diseases classified as Philadelphia chromosome-negative myeloproliferative neoplasms (Ph- MPNs), which also include chronic neutrophilic leukemia (CNL), chronic eosinophilic leukemia not otherwise specified (CEL-NOS), and unclassifiable MPN according to 2016 WHO classification (Arber et al., 2016). PV, ET, and PMF are more frequent than other subtypes of Ph- MPNs (Anderson and McMullin, 2014). Among PV, ET, and PMF, the most frequent driver mutation is *JAK2V617F*, which could be observed in the great majority of patients with PV and most patients with ET or PMF (O'Sullivan and Mead, 2019). Although sharing the same *JAK* mutation, the three *JAK2V617F* ^+^ MPN entities have distinct clinical manifestations: PV with an elevated hemoglobin/hematocrit potentially accompanied by leukocytosis or thrombocytosis, ET with thrombocytosis, and PMF with bone marrow fibrosis, extramedullary hematopoiesis, and even progressive cytopenia (Arber et al., 2016). Among these three subtypes, PMF is the more malignant and more aggressive myeloproliferative neoplasm, while PV and ET are relatively benign and indolent diseases (Grinfeld et al., 2018). However, with the progression, both PV and ET have a chance to develop into secondary myelofibrosis (SMF), sharing similar clinical features to PMF; therefore, these patients could be named post-PV-MF or post-ET-MF (Mesa et al., 2007).

The pathogenesis and progression mechanism of MPN is multifactorial. The constitutive activation of the JAK/STAT pathway due to the *JAK2V617F* acquired by the hematopoietic stem cell (HSC) plays an essential role in MPN pathogenesis (Baxter et al., 2005; Kralovics et al., 2005; Levine et al., 2005). Somatic mutations of non-driver genes related to epigenetic regulation, splicing factors, and other signaling pathways have been discovered in a large number of MPN patients, particularly in individuals with PMF (Ayalew et al., 2016; Li et al., 2016). In addition, an altered bone marrow environment and chronic inflammation could also participate in the pathogenesis and progression mechanism of MPN (Hasselbalch, 2013; Schepers et al., 2013; Bjorn and Hasselbalch, 2015; Di Buduo et al., 2015; Schmitt-Graeff et al., 2015; Zhan et al., 2018). Despite many possible causes being discovered, the underlying mechanisms of MPN onset and progression are still not entirely understood, especially in myelofibrosis.

Patients with myelofibrosis generally have an overall poor prognosis, and the median survival period is about 6 years (Rumi and Cazzola, 2017). A series of thrombohemorrhagic complications and severe constitutional symptoms are presented during disease progression. Additionally, approximately 10–20% of patients with myelofibrosis may progress to leukemic transformation (Hong et al., 2019; Vallapureddy et al., 2019). Most patients with myelofibrosis died from thrombohemorrhagic complications, cardiovascular events, and leukemic transformation (Campanelli et al., 2021).

Current treatment for myelofibrosis is still limited. Hydroxyurea (HU) has been used as first-line treatment of PMF; however, the disease course was not improved (Reilly et al., 2012). *JAK2* inhibitors such as ruxolitinib could improve the clinical symptoms of myelofibrosis (Harrison et al., 2012; Harrison et al., 2016). Unfortunately, limited molecular responses were achieved (Wang et al., 2014). Also, there are problems of drug resistance or intolerance due to long-term treatment and side effects. Allogeneic HSC transplantation is the solely possibly curative remedy for patients with myelofibrosis; however, the potential complications are more severe and complex. Therefore, it is urgently necessary to explore novel targets of myelofibrosis with the potential value of early diagnosis, prediction of progression, and accurate treatment.

Nowadays, gene expression analysis is increasingly becoming a valuable method to identify the potential targets for early diagnosis, prediction of disease progression, and treatment. Here, in this study, two gene expression datasets [GSE103237 (Zini et al., 2017) and GSE53482 (Norfo et al., 2014)] of CD34^+^ HSPCs from patients with *JAK2V617F* ^+^ MPN (PV, ET, PMF) and healthy controls (HC) were analyzed to identify important genes specifically dysregulated in *JAK2V617F* ^+^ PMF. Another two external datasets [GSE174060 (Baumeister et al., 2021) and GSE120362 (Schubert et al., 2019)] were applied to validate the results. The important genes specifically dysregulated in *JAK2V617F* ^+^ myelofibrosis might contribute to differential diagnosis and prediction of disease progression in myelofibrosis.

## Materials and methods

### Data collection

Two microarray datasets [GSE103237 (Zini et al., 2017) and GSE53482 (Norfo et al., 2014)] of patients with MPN and HC from the GEO database were retrieved. *JAK2V617F* ^+^ PV (*n* = 26), *JAK2V617F* ^+^ ET (*n* = 17), *JAK2V617F* ^+^ PMF (*n* = 23), and HC (*n* = 30) were selected for analysis. The gene expression profile from the peripheral blood/bone marrow CD34^+^ cells was annotated by the data of the GPL13667 platform.

*JAK2V617F* ^+^ PV (*n* = 8), *JAK2V617F* ^+^ ET (*n* = 2), *JAK2V617F* ^+^ SMF (*n* = 3, including 2 post-PV-MF, 1 post-ET-MF), *JAK2V617F* ^+^ PMF (*n* = 10), and HC (*n* = 6), which came from GSE174060 (Baumeister et al., 2021) and GSE120362 (Schubert et al., 2019), were used for external validation. The gene expression profile from the peripheral blood/bone marrow CD34^+^ cells was annotated by the data of the GPL17586 platform.

### Data processing and differentially expressed gene screening

The gene expression data from two datasets [GSE103237 (Zini et al., 2017) and GSE53482 (Norfo et al., 2014)] were normalized by the limma package (version 3.50.0) (Ritchie et al., 2015) in R software. Then, “ComBat” from the sva package (version 3.42.0) was used to remove batch effects after these two datasets were merged. The merged expression matrix included samples from four groups: HC, *JAK2V617F* ^+^ PV, *JAK2V617F* ^+^ ET, and *JAK2V617F* ^+^ PMF. PCA plots were performed with the Factoextra package and FactoMineR package (Lê et al., 2008; Kassambara and Mund, 2020). Then, the limma package was used to process the expression matrix and identify the DEGs between different *JAK2V617F* ^+^ MPN subtypes (PV, ET, and PMF) and HC. The adjusted *p*-value less than 0.05 and |log2 fold change (logFC)| more than 1 were regarded as significant DEGs for downstream analysis. Heatmaps were performed with the pheatmap package (Kolde, 2019). Volcano plots were generated with the ggplot2 package (Wickham, 2016). Venn plots were made with the VennDiagram package (Chen, 2021).

### Kyoto encyclopedia of genes and genomes and Reactome enrichment analysis

The clusterProfiler package (version 4.2.0) (Yu et al., 2012) was utilized to perform the Kyoto Encyclopedia of Genes and Genomes (KEGG) pathway analysis to decipher the detailed functions of DEGs in biology and disease. The clusterProfiler package (Yu et al., 2012) and ReactomePA package (version 1.38.0) (Yu and He, 2016) were used to perform Reactome pathway analysis. The significantly enriched pathways were selected with the cut-off threshold of BH adjusted *p*-value less than 0.05. The bubble diagrams of KEGG results were drawn with the enrichplot package (version 1.14.1) (Yu, 2022).

### Gene set enrichment analysis

GSEA software was utilized to run GSEA, using reference gene sets from the Molecular Signatures Database (Subramanian et al., 2005). The expression matrix of all genes from patients with MPN and HC was uploaded for analysis. The parameters of permutation number and permutation type were set as “1000” and “phenotype,” respectively. NOM *p*-value less than 0.05 was regarded as statistically significant.

### Analysis of protein–protein interaction networks and identification of hub genes

Protein–protein interaction (PPI) analysis of DEGs was constructed based on the STRING database (Szklarczyk et al., 2015). Cytoscape (Shannon et al., 2003) was utilized to further process the results from PPI networks. The cytoHubba app (Chin et al., 2014) was utilized for obtaining the hub DEGs (top ten DEGs) by the MCC algorithm, and the MCODE app (Bader and Hogue, 2003) was applied to identify the sub-networks (top two modules).

### External validation of differentially expressed genes

Another two datasets [GSE174060 (Baumeister et al., 2021) and GSE120362 (Schubert et al., 2019)] from the GEO database were retrieved. Samples, including HC (*n* = 6), *JAK2V617F* ^+^ PV (*n* = 8), *JAK2V617F* ^+^ ET (*n* = 2), *JAK2V617F* ^+^ SMF (*n* = 3), and *JAK2V617F* ^+^ PMF (*n* = 10), were selected for external validation. The gene expression matrix of CD34^+^ HSPCs from bone marrow/peripheral blood was annotated by the GPL17586 platform data. The pipeline of data processing is similar to the methods described above. The expression levels of DEGs between patients with MPN and HC were compared with Wilcoxon rank-sum test from the ggpubr package (version 0.4.0) (Kassambara, 2020). The *p* value less than 0.05 was regarded as having statistical significance. The GraphPad Prism (version 9.3.1) was used to calculate the value of AUC and draw the ROC curve.

### Statistical analysis

R software and GraphPad Prism were utilized for data processing and statistical analysis. The ggpubr package in R software was applied to draw boxplots and perform statistical analysis. The Wilcoxon rank-sum test was applied to compare the difference in expression levels between distinct groups. The *p* value less than 0.05 at two-sided was regarded as statistically significant. “*” denotes “*p* < 0.05”, “**” denotes “*p* < 0.01”, “***” denotes “*p* < 0.001”, “****” denotes “*p* < 0.0001”, and “ns” denotes “not significant”.

## Results

### CD34^+^ hematopoietic stem and progenitor cells from *JAK2V617F* ^+^ primary myelofibrosis exhibited a greater number of dysregulated genes than those from *JAK2V617F* ^+^ polycythemia vera and *JAK2V617F* ^+^ essential thrombocythemia

**FIGURE 1:**
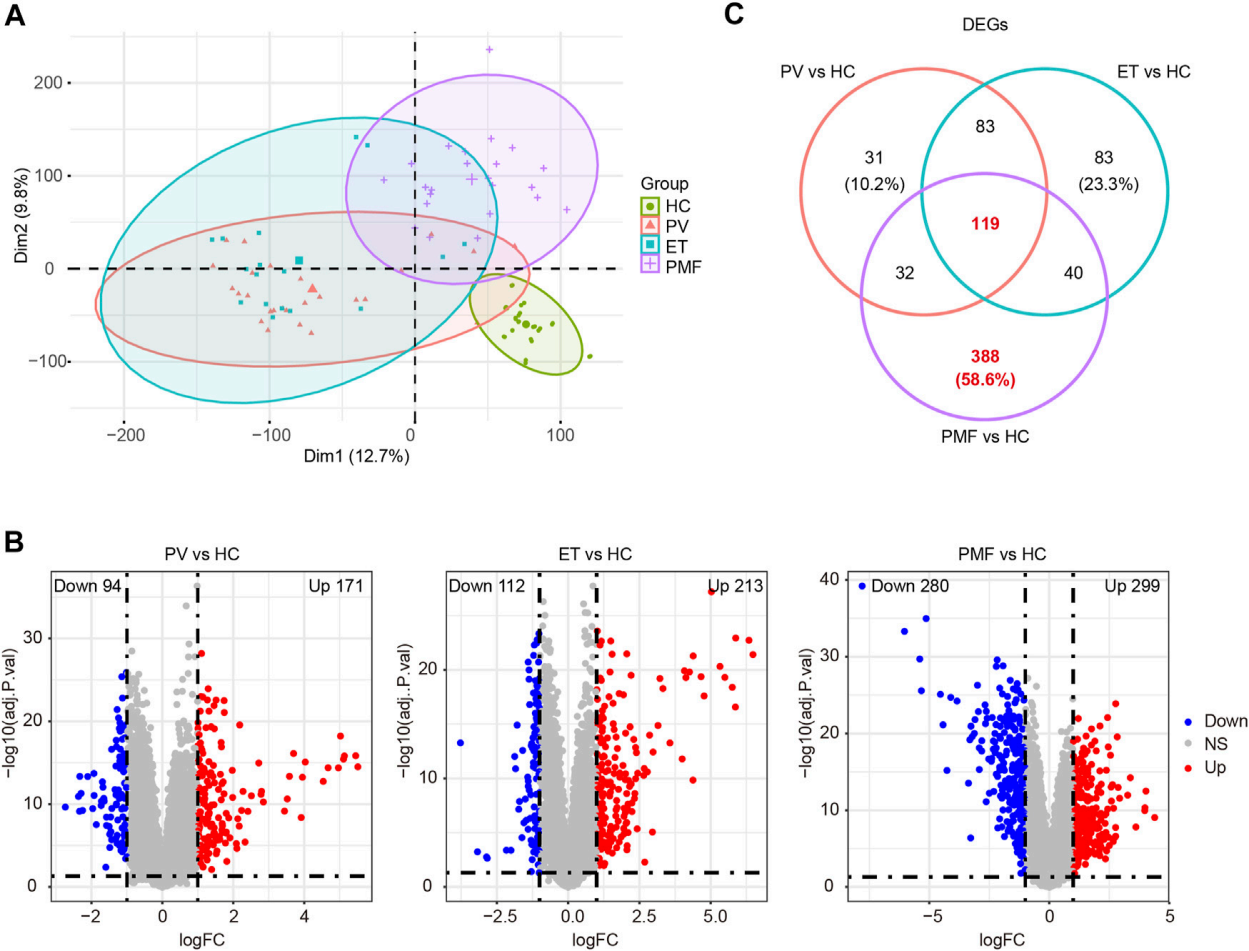
**(A)** PCA diagram after removing batch effect. **(B)** Volcano plots of DEGs between each MPN subtype and healthy controls (HC). The numbers of upregulated genes and downregulated genes were shown. The red dots represent upregulated genes. The blue dots indicate downregulated genes. The gray dots denote genes with non-differential expression. **(C)** Venn diagram of DEGs between each MPN subtype and HC.

A total of 96 samples, including HC (*n* = 30), *JAK2V617F* ^+^ PV (*n* = 26), *JAK2V617F* ^+^ ET (*n* = 17), and *JAK2V617F* ^+^ PMF (*n* = 23), were collected from two datasets [GSE103237 (Zini et al., 2017) and GSE53482 (Norfo et al., 2014)] based on the same GPL platform. After removing the batch effect, the PCA result showed that the features of three *JAK2V617F* ^+^ MPN subtypes could be distinguishable from those of HC (Figure 1A), suggesting the signatures of HSPCs under disease state changed significantly compared to those in normal state. In addition, the features of *JAK2V617F* ^+^ PV and ET were similar to each other but distinct from *JAK2V617* ^+^ PMF (Figure 1A), which might be explained by that they were indeed in the different stages (“indolent” and “aggressive” stages, respectively) of MPN. Furthermore, we also noticed that the features from patients with three *JAK2V617F* ^+^ MPN entities were less uniform than those of HC (Figure 1A), indicating the heterogeneity of CD34^+^ HSPCs existed not only between different MPN subtypes but also in the same MPN subtype.

Next, the gene expression matrix of *JAK2V617F* ^+^ PV (*n* = 26) and HC (*n* = 30) was processed to discover the differentially expressed genes (DEGs). A total of 265 DEGs were identified from the matrix, including 171 upregulated genes and 94 downregulated genes in patients with *JAK2V617F* ^+^ PV compared to HC (Figure 1B). A similar DEG screening procedure was also performed in *JAK2V617F* ^+^ ET (*n* = 17) and *JAK2V617F* ^+^ PMF (*n* = 23). A total of 213 upregulated DEGs and 112 downregulated DEGs were found in patients with *JAK2V617F* ^+^ ET relative to HC (Figure 1B), and 299 upregulated DEGs and 280 downregulated DEGs were obtained in patients with *JAK2V617F* ^+^ PMF relative to HC (Figure 1B). The number of dysregulated genes identified from PMF was much more abundant than that from PV and ET, indicating the more complex and severe disease course of PMF.

Integration of all DEGs between each MPN subtype and HC showed that among the different MPN subtypes, the DEG expression patterns from *JAK2V617F* ^+^ PV and ET patients were distinct from those from PMF patients (Supplementary Figures S1A–D). However, Partial patterns shared by the three entities were also observed (Supplementary Figures S1A–D). These shared and different signatures might provide important clues for the pathogenesis and heterogeneity of MPN. Therefore, the overlapping or unique DEGs derived from the integrated matrix were identified (Figure 1C and Supplementary Table S1). A total of 119 overlapping DEGs in patients with three different MPN subtypes were obtained, and 31 DEGs were merely acquired in *JAK2V617F* ^+^ PV, while 83 DEGs were uniquely obtained in *JAK2V617F* ^+^ ET (Figure 1C). Among three MPN entities, *JAK2V617F* ^+^ PMF had the greatest number of specific DEGs, with 388 DEGs (58.6%) exclusively dysregulated in PMF (Figure 1C). These specific dysregulated genes might play an essential part in the onset and progression of myelofibrosis.

In summary, the results corroborated different patterns of “mild and indolent subtype” and “aggressive and fibrotic subtype” in *JAK2V617F* ^+^ MPN, suggesting that the heterogeneity of HSPCs is obvious between different MPN subtypes and *JAK2V617F* ^+^ PMF might have more complicated pathogenesis than PV or ET.

### CD34^+^ hematopoietic stem and progenitor cells from *JAK2V617F* ^+^ primary myelofibrosis enriched a larger number of inflammatory pathways and immune response signaling pathways than those from *JAK2V617F* ^+^ polycythemia vera and *JAK2V617F* ^+^ essential thrombocythemia

**FIGURE 2:**
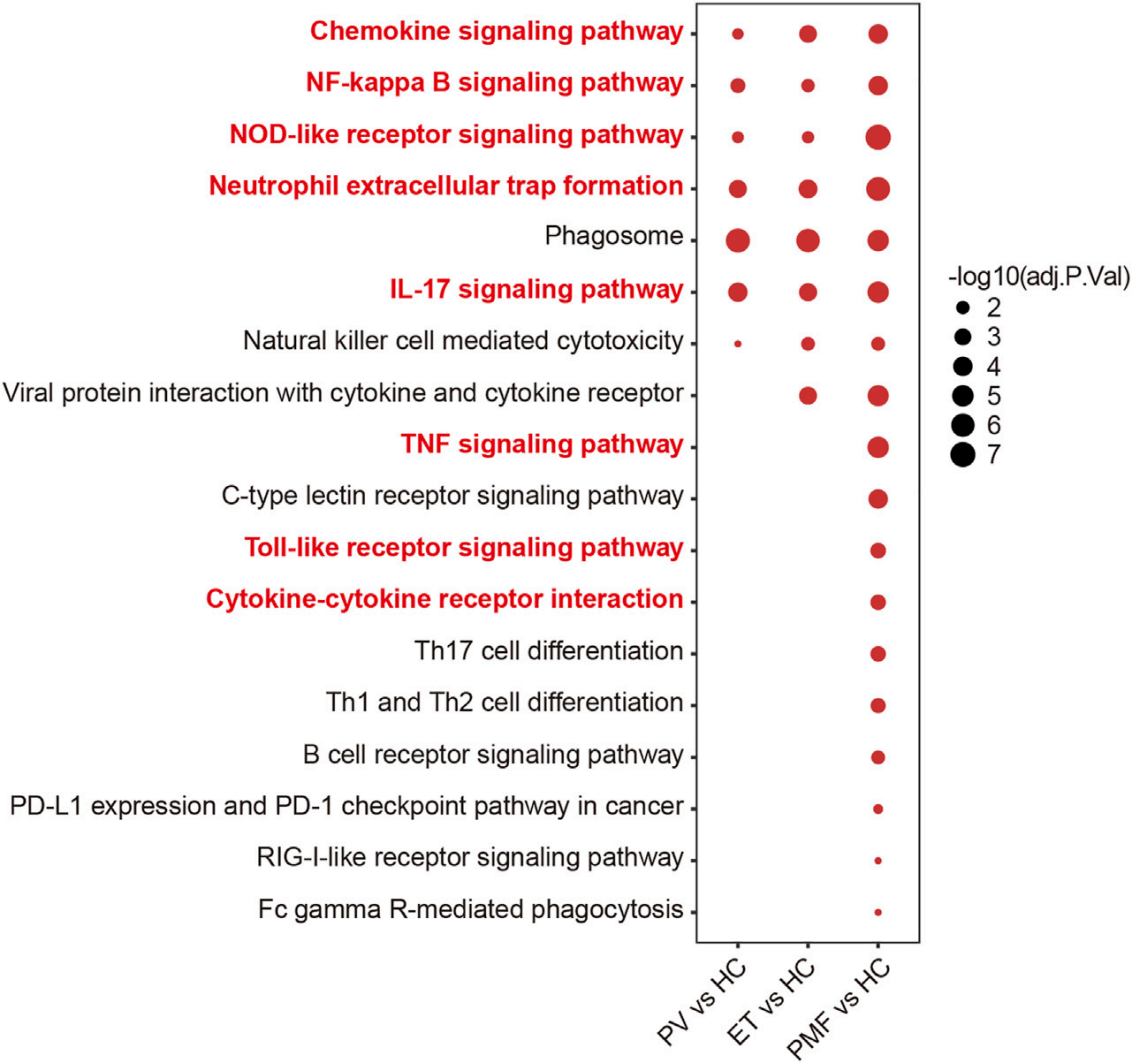
Inflammation- and immune-related pathways from KEGG analysis of upregulated genes in each MPN subtype compared to HC. The size of the dots represents −log10 (adjusted *p* value).

The KEGG database was utilized to investigate the detailed function of DEGs and identify the shared and distinct enriched pathways between three MPN subtypes. Considering the upregulated genes were more potentially prominent in biological function than the downregulated genes, we focused on the upregulated genes in each MPN subtype compared to HC to perform the downstream pathway enrichment analysis. The results suggested that most upregulated genes were enriched mainly in pathways correlated with immune response and inflammation. Some of the inflammatory pathways and immune response signaling pathways were enriched in three MPN subtypes, such as chemokine signaling pathway, NF-kappa B signaling pathway, NOD-like receptor signaling pathway, neutrophil extracellular trap formation, phagosome, as well as IL-17 signaling pathway (Figure 2), demonstrating that the inflammation and related immune response may participate in the pathogenesis of three MPN entities. However, the number of these enriched pathways in PMF was obviously more than that in PV and ET. For example, the TNF signaling pathway, toll-like receptor signaling pathway, and cytokine–cytokine receptor interaction were also enriched in PMF, but not in PV or ET (Figure 2). The results suggested that the more broad and severe state of inflammation and immune response might contribute to the aggressive progression of myelofibrosis.

**FIGURE 3:**
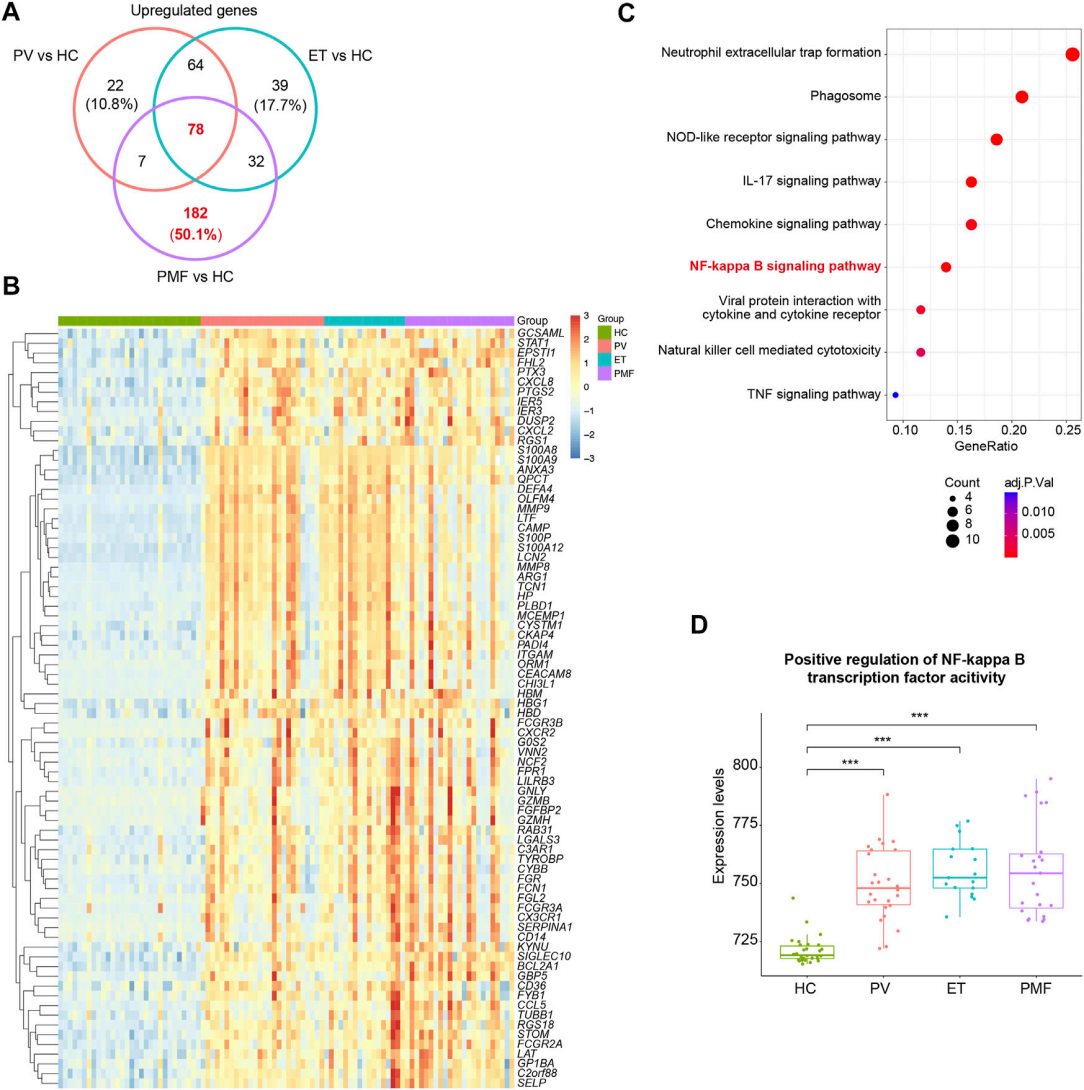
**(A)** Venn diagram of upregulated genes in each MPN subtype. **(B)** Heatmap of overlapping upregulated genes in three MPN subtypes (*JAK2V617F* ^+^ PV, *JAK2V617F* ^+^ ET, and *JAK2V617F* ^+^ PMF). **(C)** Inflammation- and immune-related pathways from KEGG analysis of the overlapping upregulated genes in three MPN subtypes. The size of the dots represents the number of genes. The color of the dots denotes the adjusted *p* value. **(D)** Expression levels of the gene set named “Positive regulation of NF-kappa B transcription factor activity” in three MPN subtypes and HC.

To further discover the potential biological function of upregulated genes, we performed the venn diagram and identified the overlapping and unique upregulated genes among three MPN subtypes (Figure 3A and Supplementary Table S1). The results showed the number of upregulated genes uniquely in PMF was much more than that exclusively in PV or ET and even more than the number of overlapping genes (Figure 3A), suggesting that beyond some common factors underlying the pathogenesis, such as inflammation previously mentioned, there were additional elements leading to myelofibrosis. Considering the fundamental and essential roles of the common factors involved in the pathogenesis of three MPN subtypes, we studied these common characteristics before identifying the heterogeneous features. The overlapping upregulated genes (*n* = 78) were shown (Figure 3B), and KEGG analysis indicated that most of them were enriched in inflammatory pathways and immune response pathways, such as the NF-kappa B signaling pathway (Figure 3C). The expression levels of the relative gene set and the GSEA results between each MPN subtype and HC also corroborated the upregulation of the NF-kappa B signaling pathway in distinct MPN subtypes (Figure 3D, Supplementary Figure S2).

In summary, these results indicated that inflammation and immune response constitute the fundamental and essential parts of the pathogenesis in MPN, and the difference in degree and scope of them might be one of the explanations for more severe manifestation in myelofibrosis.

### The overlapping upregulated genes were identified in distinct *JAK2V617F* ^+^ myeloproliferative neoplasm subtypes and primarily related to the inflammatory pathways and immune response signaling pathways

**FIGURE 4:**
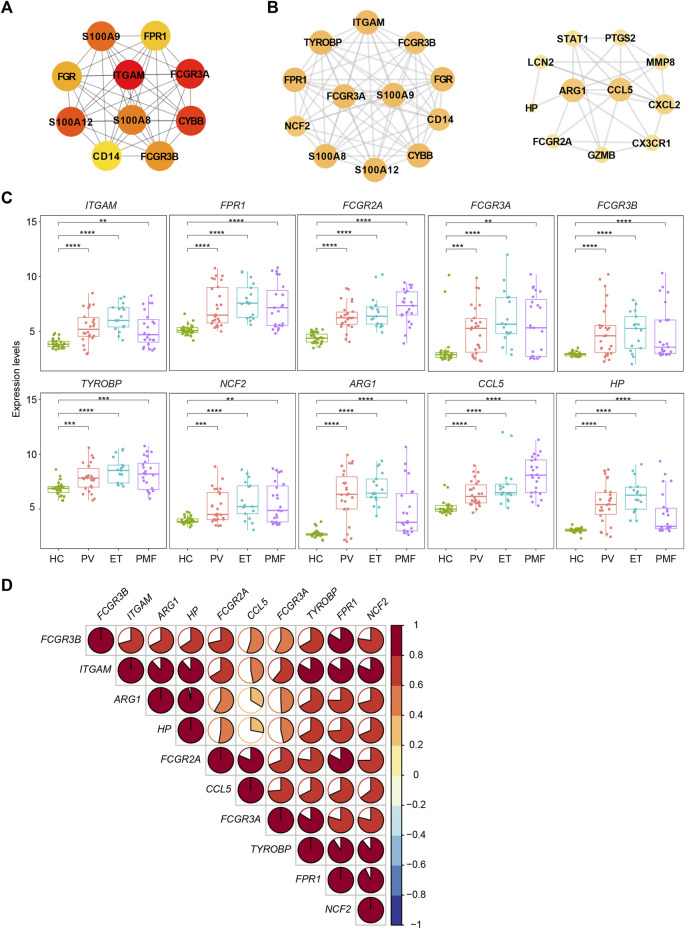
**(A)** Top 10 hub genes from the PPI network analysis for the overlapping upregulated genes in three MPN subtypes. Nodes denote encoded proteins, and edges denote the interaction between encoded proteins. The color represents the scores ranked by the MCC method. The deeper color denotes the more important genes having higher scores. **(B)** Top two significant modules from the PPI network analysis for the overlapping upregulated genes in three MPN subtypes. The color and size represent the degrees of the nodes. The bigger size and deeper color denote the more important genes having higher degrees. **(C)** Expression levels of the inflammation- and immune-related genes upregulated in three MPN subtypes. **(D)** Correlation of the expression levels of the upregulated genes in **(C)**.

To resolve the interaction between proteins encoded by overlapping upregulated genes (*n* = 78) in three *JAK2V617F* ^+^ MPN subtypes, the STRING database was utilized to conduct the analysis. The results indicated that, in total, 405 edges (protein interaction pairs) and 78 nodes (encoded proteins) were included. Ten hub genes of overlapping upregulated genes in three *JAK2V617F* ^+^ MPN subtypes were obtained by the cytoHubba plugin from the PPI network (Figure 4A). Also, the hub genes, such as *ITGAM*, *FPR1*, Fc Gamma Receptor genes (*FCGR3A* and *FCGR3B*), and S100 family genes (*S100A8/9/12*), were related to inflammatory pathways and immune response signaling pathways (Figure 4A). In addition, two significant modules were acquired by the MCODE plugin, module 1 containing 12 nodes and 64 edges and module 2 containing 11 nodes and 30 edges, including genes related to immune response signaling pathways, such as *FPR1*, *NCF2*, *ARG1*, *CCL5*, and *HP* (Figure 4B). Furthermore, we found that the expression levels of some identified genes (*ITGAM*, *FPR1*, Fc Gamma Receptor genes, *TYROBP*, *NCF2*, *ARG1*, *CCL5*, and *HP*) in three *JAK2V617F* ^+^ MPN subtypes were significantly higher than those in HC (Figure 4C). And, most of their expression levels were also positively correlated with each other (Figure 4D).

**FIGURE 5:**
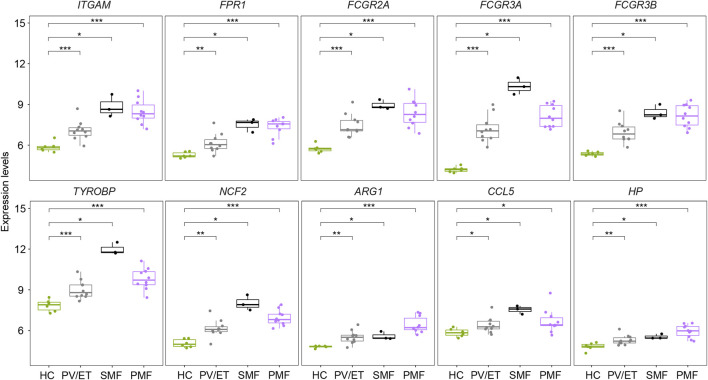
Expression levels of the inflammation- and immune-related genes in distinct MPN subtypes and HC from the external datasets.

Two external datasets [GSE174060 (Baumeister et al., 2021) and GSE120362 (Schubert et al., 2019)] derived from the same GPL platform were utilized and analyzed to verify the inflammation- and immune-related genes overlapping upregulated in different *JAK2V617F* ^+^ MPN subtypes, with CD34^+^ HSPC samples of *JAK2V617F* ^+^ PV (*n* = 8), *JAK2V617F* ^+^ ET (*n* = 2), *JAK2V617F* ^+^ SMF (*n* = 3), *JAK2V617F* ^+^ PMF (*n* = 10), and HC (*n* = 6) included. Due to the paucity of CD34^+^ samples in patients with *JAK2V617F* ^+^ ET, we integrated the samples of *JAK2V617F* ^+^ PV and *JAK2V617F* ^+^ ET into one group named *JAK2V617F* ^+^ PV/ET, reflecting the chronic and indolent stage of MPN. The expression levels of these inflammation- and immune-related genes (*ITGAM*, *FPR1*, Fc Gamma Receptor genes, *TYROBP*, *NCF2*, *ARG1*, *CCL5*, and *HP*) were significantly higher in both chronic and aggressive fibrotic phases of *JAK2V617F* ^+^ MPN than those in HC (Figure 5), which confirmed the results described above. Taken together, these results suggested that the upregulation of these genes associated with inflammation and immune response might play fundamental roles in the pathogenesis of MPN.

### Interferon signaling pathways were upregulated significantly and specifically in CD34^+^ hematopoietic stem and progenitor cells of *JAK2V617F* ^+^ primary myelofibrosis

**FIGURE 6:**
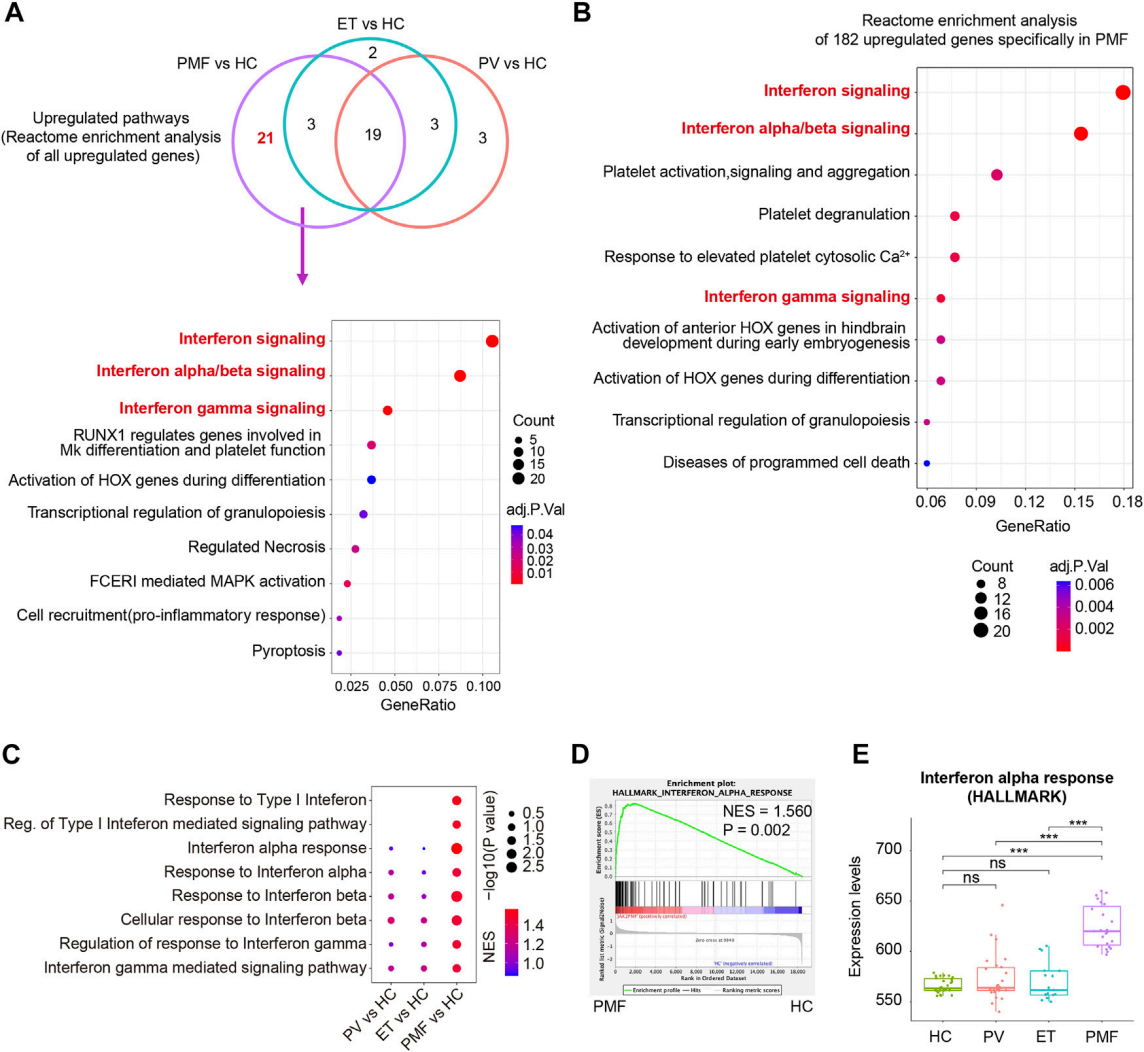
**(A)** Venn diagram of enriched Reactome pathways of upregulated genes in each MPN subtype compared to HC. Some of the 21 enriched Reactome pathways uniquely in *JAK2V617F* ^+^ PMF were shown in the dot plot. The size of the dots represents the number of genes. The color of the dots denotes the adjusted *p* value. **(B)** Top 10 enriched Reactome pathways of 182 upregulated genes uniquely in *JAK2V617F* ^+^ PMF were shown in the dot plot. The size of the dots represents the number of genes. The color of the dots denotes the adjusted *p* value. **(C)** Gene set enrichment analysis (GSEA) of the Interferon-related signaling pathways between each MPN subtype and HC. The size of the dots represents −log10 (NOM *p* value). The color of the dots denotes the NES. **(D)** GSEA of the gene set “Interferon alpha response” between *JAK2V617F* ^+^ PMF and HC. **(E)** Expression levels of the gene set “Interferon alpha response” in three MPN subtypes and HC.

To further explore the potential detailed function of upregulated genes uniquely in PMF and identify the **s**ignaling pathways solely enriched in myelofibrosis, the Reactome database including more various signaling pathway information was used to resolve the roles of upregulated genes in each MPN subtype compared to HC. The Reactome enrichment analysis identified the overlapping enriched pathways (*n* = 19) (Figure 6A, Supplementary Table S2), most of which were related to inflammation and immune response, indicating the identical conclusion to the analysis described above. More importantly, we identified the uniquely enriched signaling pathways (*n* = 21) in PMF (Figure 6A, Supplementary Table S2). Interestingly, we surprisedly found that interferon signaling pathways were enriched exclusively in CD34^+^ HSPCs of PMF, but not in those of PV or ET (Figure 6A). In addition, when we selected the upregulated genes (*n* = 182) uniquely in PMF to perform the Reactome enrichment analysis, interferon signaling pathways were also enriched significantly, such as interferon signaling, interferon-alpha/beta signaling, and interferon-gamma signaling (Figure 6B). Furthermore, the GSEA analysis was conducted, and the result also showed that multiple gene sets of interferon signaling pathways were enriched significantly and exclusively in CD34^+^ HSPCs of *JAK2V617F* ^+^ PMF, but not in those of PV or ET (Figures 6C,D), corroborating the previous results. The expression levels of gene set “interferon alpha response” also verified that the upregulation of the interferon signaling pathway was significantly and specifically in *JAK2V617F* ^+^ PMF (Figure 6E).

Therefore, the upregulation of interferon signaling pathways might participate in the pathogenesis of myelofibrosis.

### Four interferon-stimulated genes (*OAS1*, *IFITM3*, *GBP1*, and *GBP2*) were upregulated specifically in CD34^+^ hematopoietic stem and progenitor cells of myelofibrosis

**FIGURE 7:**
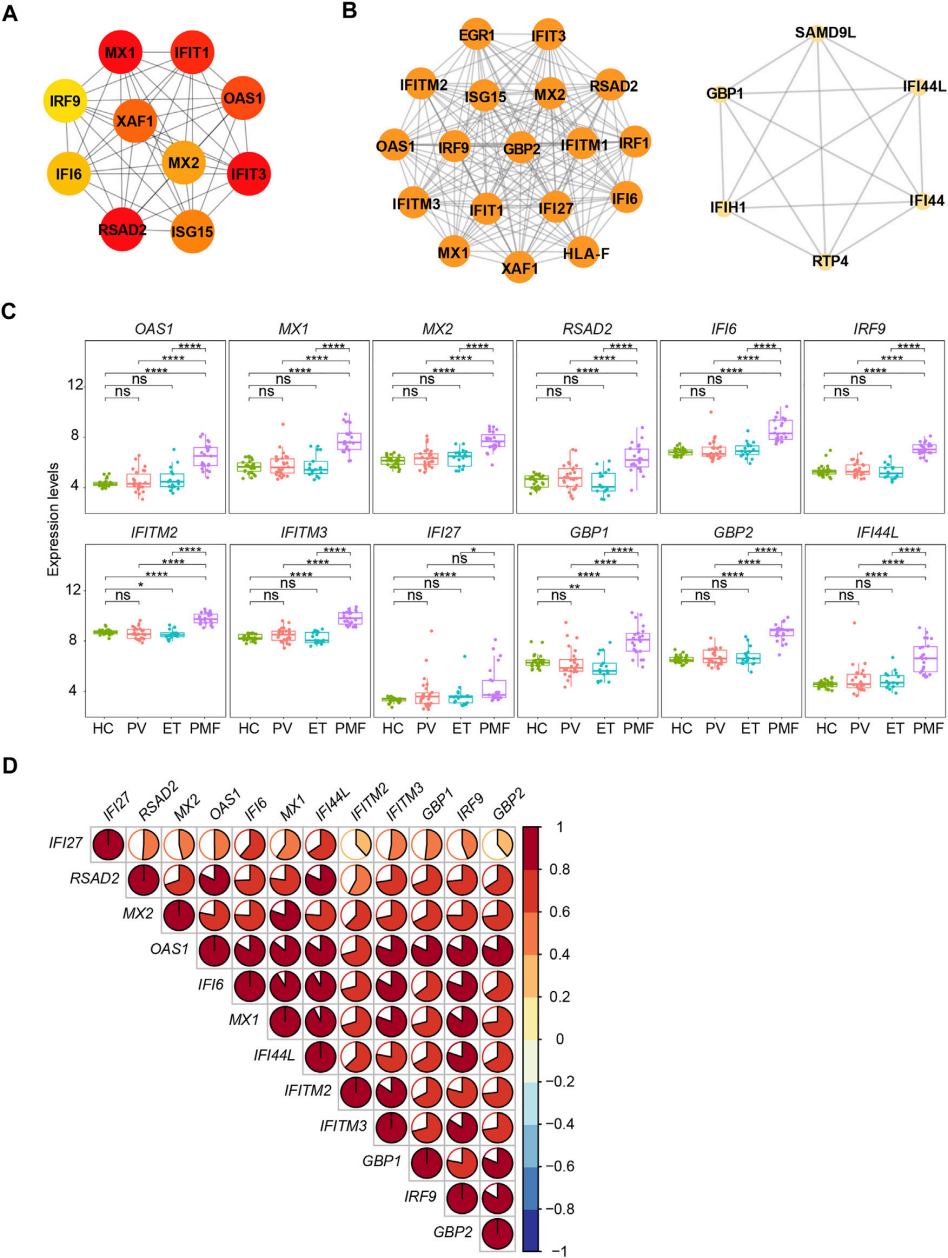
**(A)** Top 10 hub genes from the PPI network analysis for the upregulated genes uniquely in *JAK2V617F* ^+^ PMF. Nodes denote encoded proteins, and edges denote the interaction between two encoded proteins. The color represents the scores ranked by the MCC method. The deeper color denotes the more important genes having higher scores. **(B)** Top two significant modules from the PPI network analysis for the upregulated genes uniquely in *JAK2V617F* ^+^ PMF. The color and size represent the degrees of the nodes. The bigger size and deeper color denote the more important genes having higher degrees. **(C)** Expression levels of the key genes uniquely upregulated in *JAK2V617F* ^+^ PMF. **(D)** Correlation of the expression levels of genes in **(C)**.

The PPI network of these upregulated DEGs (*n* = 182) exclusively in *JAK2V617F* ^+^ PMF was constructed with 179 nodes (encoded proteins) and 322 edges (protein interaction pairs). Through the PPI network, the top 10 hub genes were discovered, namely, *MX1*, *IFIT1*, *IFIT3*, *OAS1*, *RSAD2*, *XAF1*, *MX2*, *ISG15*, *IFI6*, and *IRF9*, most of which were strongly associated with interferon-gamma signaling (Figure 7A). Two significant modules were also discovered (Figure 7B). Module 1 had 18 nodes and 153 edges, and module 2 contained 6 nodes and 15 edges, and the involved genes were almost related to the interferon signaling pathway (Figure 7B). Additionally, we found that the interferon-related genes (*OAS1*, *MX1*, *MX2*, *RSAD2*, *IFI6*, *IRF9*, *IFITM2*, *IFITM3*, *GBP1*, *GBP2*, and *IFI44L*) were upregulated in *JAK2V617F* ^+^ PMF compared to not only HC but also *JAK2V617F* ^+^ PV and ET (Figure 7C). Furthermore, there was a positive correlation in the expression levels between most genes (Figure 7D).

**FIGURE 8:**
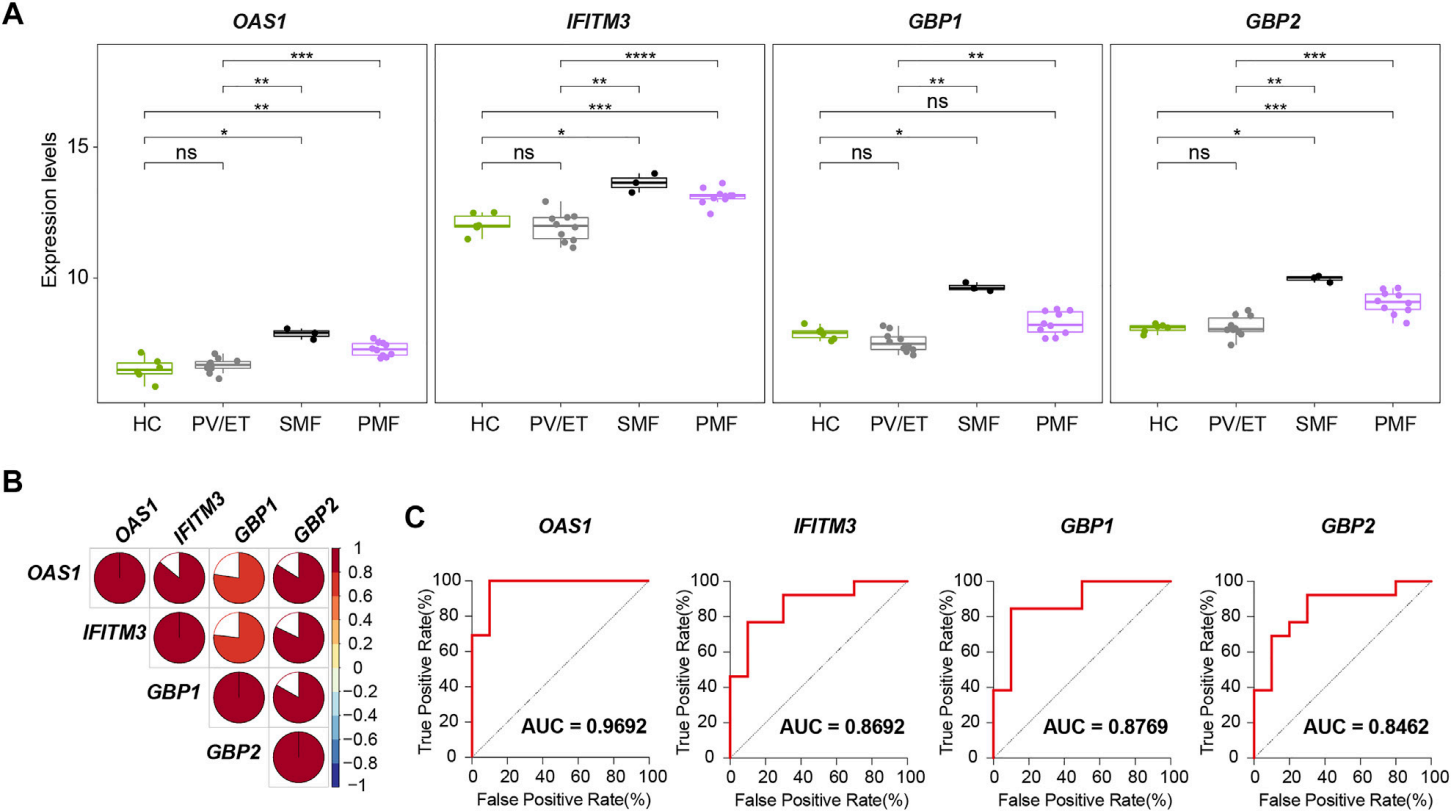
**(A)** Expression levels of four key genes in distinct MPN subtypes and HC from the external datasets. **(B)** Correlation of the expression levels of the four key genes in **(A)**. **(C)** ROC curves of the four key genes for the diagnosis of *JAK2V617F* ^+^ myelofibrosis in the external datasets.

Two external datasets [GSE174060 (Baumeister et al., 2021) and GSE120362 (Schubert et al., 2019)] described above were used as validation datasets. Considering the additional three samples from patients with *JAK2V617F* ^+^ SMF, we finally identified four genes (*OAS1*, *IFITM3*, *GBP1*, and *GBP2*) with increased expression levels not only in *JAK2V617F* ^+^ PMF but also in *JAK2V617F* ^+^ SMF in comparison with HC and PV/ET (Figure 8A). In addition, their expression levels are also positively correlated well with each other (Figure 8B).

To investigate the differential diagnostic ability of the four genes (*OAS1*, *IFITM3*, *GBP1*, and *GBP2*) for patients with myelofibrosis, the ROC curves and AUC were performed. In the ROC curves here, the true positive rate means the percentage of that a *JAK2V617F* ^+^ myelofibrosis patient is correctly predicted as *JAK2V617F* ^+^ myelofibrosis, while the false positive rate is the proportion of that a *JAK2V617F* ^+^ PV or ET individual is classified falsely as *JAK2V617F* ^+^ myelofibrosis. The AUC represents the area under the ROC curve, indicating the accuracy and reliability of diagnostic prediction. The results showed high AUC values of the four genes (*OAS1*, *IFITM3*, *GBP1*, and *GBP2*) when distinguishing *JAK2V617F* ^+^ myelofibrosis from *JAK2V617F* ^+^ PV or ET (Figure 8C). Among the four genes, *OAS1* had the highest AUC value. Therefore, these four genes (*OAS1*, *IFITM3*, *GBP1*, and *GBP2*), especially *OAS1*, might possess the potential to be novel auxiliary diagnostic and predictive indicators of myelofibrosis, but further research is still necessary in the future.

## Discussion

Although abundant research about Ph- MPNs has been performed, the mechanisms of disease onset and progression in myelofibrosis are still not fully understood. Considering there are significantly diverse manifestations and prognoses between the chronic stage and aggressive stage of MPN, it is urgently needed to identify some reliable biomarkers to predict myelofibrosis as early as possible, which could also contribute to early intervention and treatment. Here, we precisely analyzed the gene expression profiles in different subtypes of *JAK2V617F* ^+^ MPN (including PV, ET, SMF, and PMF) to provide new information on pathogenesis and identify novel indicators for diagnosis and prediction of myelofibrosis.

Inflammation could be an important determinant that promotes the development and progression of MPN. Recent studies found that the acquisition of *JAK2V617F* in HSPCs, leading to the cytokine-independent activation of JAK-STAT signaling, may occur even during early childhood and *in utero*, and there is a very long asymptomatic period (Van Egeren et al., 2021; Williams et al., 2022). Chronic inflammation might be one required factor for transformation from asymptomatic clonal hematopoiesis to overt MPN or even for the progression from PV/ET to myelofibrosis. Studies about inflammatory cytokines indicated that the levels of IL6, IL2, and sIL2a were elevated during the progression from PV/ET to myelofibrosis (Panteli et al., 2005). Here, we also observed the upregulated pathways and genes strongly associated with inflammation and immune response. For instance, the chemokine signaling pathway, NF-kappa B signaling pathway, as well as pro-inflammatory genes (such as S100 family genes) were upregulated in three *JAK2V617F* ^+^ MPN subtypes, consistent with some published findings (Fisher et al., 2017; Fisher et al., 2019; Baumeister et al., 2021). Some inflammation- and immune-related upregulated genes (*ITGAM*, *FPR1*, Fc Gamma Receptor genes, *TYROBP*, *NCF2*, *ARG1*, *CCL5*, and *HP*), which were undetected in previously published studies, were also identified in distinct *JAK2V617F* ^+^ MPN subtypes in comparison with HC. Additionally, among three subtypes, *JAK2V617F* ^+^ PMF has more numbers of inflammation-related upregulated pathways and genes, suggesting that the more prominent inflammatory condition is a pronounced feature of myelofibrosis.

Furthermore, we identified four potentially important genes (*OAS1*, *IFITM3*, *GBP1*, and *GBP2*) that were upregulated uniquely in myelofibrosis. These genes belong to interferon-stimulated genes, related closely to interferon signaling (Schneider et al., 2014). Previous studies suggested that interferon (IFN) and IFN-stimulated genes might be involved in pulmonary fibrosis (Neville et al., 1997; Berkmana et al., 2001; Christmann et al., 2014). The low dose of IFN-α could promote bleomycin-induced lung fibrosis in mice and hamsters, though this effect was complicated and might also be influenced by IFN preparations (Neville et al., 1997; Berkmana et al., 2001). The expression levels of interferon-regulated genes (*OAS1* and *IFI44*) were found to be positively correlated with progressive lung fibrosis in patients with systemic sclerosis (SSc)–related interstitial lung disease (ILD) (Christmann et al., 2014). Additionally, a recent study demonstrated that a high expression level of *IFITM3* could reflect the adverse prognosis in AML (Liu et al., 2020). However, the roles of IFN and interferon-stimulated genes in the pathogenesis of myelofibrosis are still largely unclear. Our previously published research showed that *JAK2* mutant HSCs displayed increased IFN signaling, which might promote Mk (megakaryocyte)-biased hematopoiesis (Tong et al., 2021). There was another study showing that *GBP2* overexpression suppressed the erythroid differentiation and increased the level of matrix metalloproteinase-9 in TF-1 cells (Lin et al., 2013), which was also found to be increased in patients with idiopathic myelofibrosis (Xu et al., 2005). These results suggested the characteristics including thrombocythemia, anemia, and even bone marrow failure in myelofibrosis might be related to the increased IFN signaling, but more evidence should be required in the future. Overall, the four interferon-stimulated genes (*OAS1*, *IFITM3*, *GBP1*, and *GBP2*) exclusively upregulated in myelofibrosis might not only provide important novel clues to the MPN field but also offer special insights into the effects of IFN signaling on the pathogenesis of myelofibrosis.

Inevitably, this study has limitations. Though quite large numbers of samples and repeated validations from multiple datasets were applied to minimalize the individual variation, this study was limited by the lack of detailed clinical information in these GEO datasets such as variant allele frequency (VAF) of *JAK2V617F*, other somatic mutations, disease duration, and treatment, which remains to be further investigated.

In summary, our study precisely analyzed the gene expression profiles of CD34^+^ HSPCs across the different *JAK2V617F* ^+^ MPN subtypes. The gene expression signatures in *JAK2V617F* ^+^ PMF were to a large extent distinct from those in *JAK2V617F* ^+^ PV or ET, although there were also similar features among them. The upregulation of inflammation- and immune-related signature genes and especially IFN signaling might be involved in the pathogenesis and progression of myelofibrosis. Additionally, the four genes (*OAS1*, *IFITM3*, *GBP1*, and *GBP2*) we identified in this study might have the potential to be auxiliary diagnostic and predictive indicators of myelofibrosis, but further investigations are still necessary in the future.

## Data Availability

Publicly available datasets were analyzed in this study. The datasets presented in this study can be found in GEO database (https://www.ncbi.nlm.nih.gov/geo/). The accession numbers of the datasets can be found in the article/Supplementary Material.

## Supplementary material

Supplementary Figure S1
**(A)** Heatmap of all DEGs between each MPN subtype and HC. The blue color indicates the downregulated genes, and the red color denotes the upregulated genes. **(B)** Heatmap of top 20 upregulated genes between *JAK2V617F* ^+^ PV and HC. **(C)** Heatmap of top 20 upregulated genes between *JAK2V617F* ^+^ ET and HC. **(D)** Heatmap of top 20 upregulated genes between *JAK2V617F* ^+^ PMF and HC.

Supplementary Figure S2 GSEA of the gene set “Positive regulation of NF-kappa B transcription factor activity” between each MPN subtype and HC.

Supplementary Table S1
**(A)** DEGs commonly or uniquely dysregulated in *JAK2V617F* ^+^ PV, *JAK2V617F* ^+^ ET or *JAK2V617F* ^+^ PMF. **(B)** DEGs commonly or uniquely upregulated in *JAK2V617F* ^+^ PV, *JAK2V617F* ^+^ ET or *JAK2V617F* ^+^ PMF.

Supplementary Table S2 Reactome pathways commonly or uniquely enriched in *JAK2V617F* ^+^ PV, *JAK2V617F* ^+^ ET, or *JAK2V617F* ^+^ PMF.
