# Supplementary figures and images for: Expression profiles analysis identifies specific interferon-stimulated signatures as potential diagnostic and predictive indicators of JAK2V617F + myelofibrosis
Source: Front Genet. 2022 Aug 18;13:927018. doi: 10.3389/fgene.2022.927018 (PMC9434717; doi:10.3389/fgene.2022.927018)

Figure S1

A

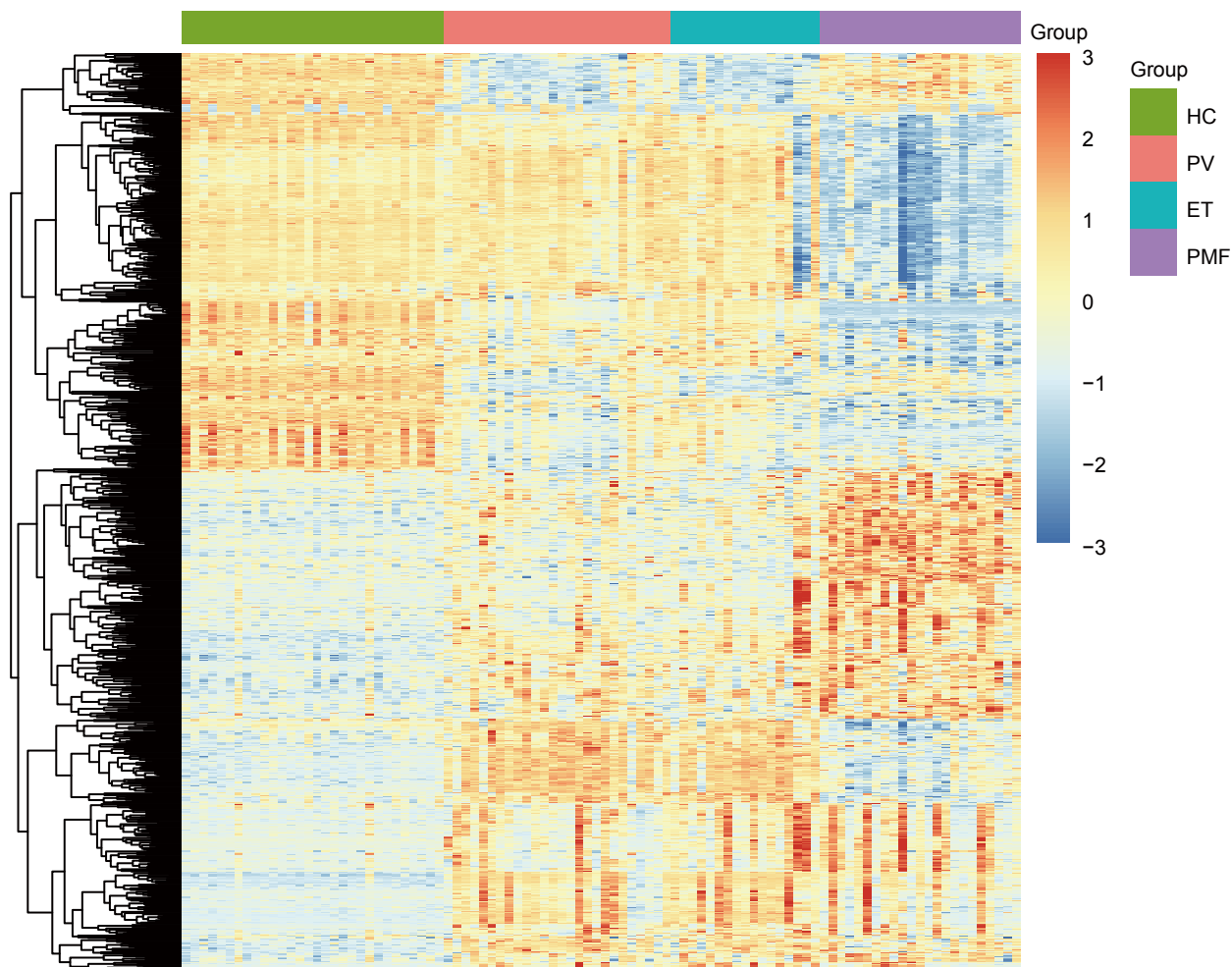

B

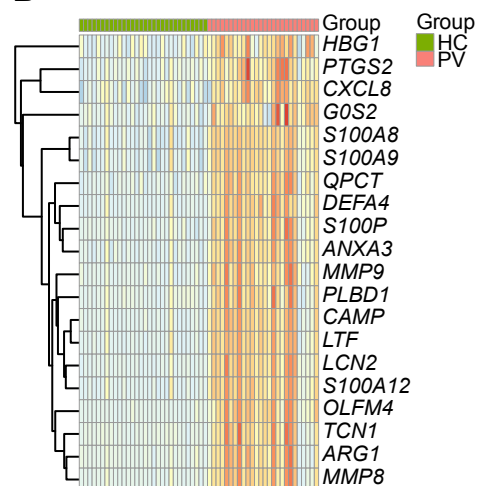

C

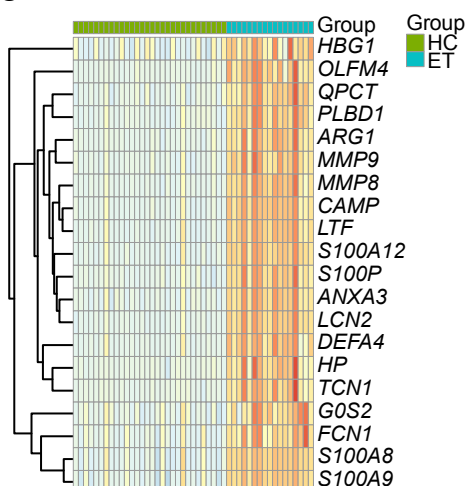

D

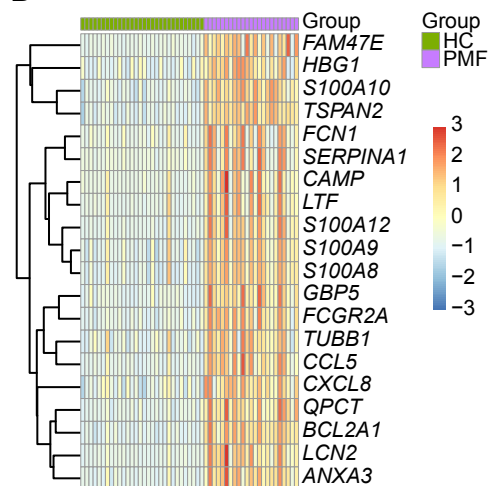

Figure S2

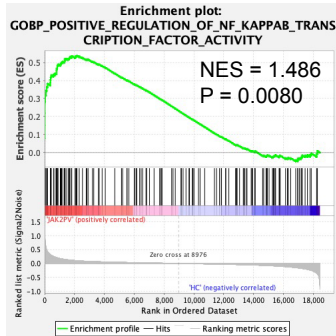

PV

HC

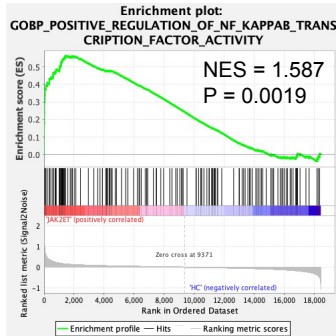

ET

HC

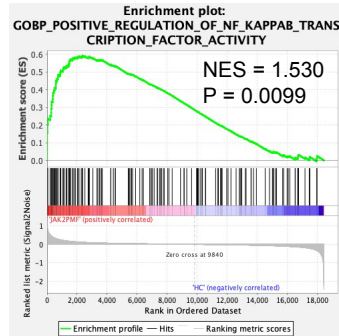

PMF

HC

Supplement: Supplementary file 2 [file DataSheet1.pdf]
